# Atrial Fibrillation at Hassan II Regional Hospital, Agadir, Morocco: Insights Into Presentation and Management

**DOI:** 10.7759/cureus.98986

**Published:** 2025-12-11

**Authors:** Mehdi Berrajaa, Hala Jaouhari, Wassim Beladel, Khalil Abderrahmane Elbaz, Mohamed El Minaoui

**Affiliations:** 1 Cardiology Department, Souss-Massa University Hospital, Faculty of Medicine and Pharmacy, Ibn Zohr University, Agadir, MAR

**Keywords:** anticoagulants, atrial fibrillation, epidemiology, morocco, retrospective studies

## Abstract

Background

Atrial fibrillation (AF) is a common supraventricular arrhythmia marked by chaotic, uncoordinated atrial activity that impairs effective contraction. This hemodynamic inefficiency promotes intracardiac thrombus formation, making AF a major cause of ischemic cerebral events and other embolic events.

Objectives

The objective of our study is to describe the epidemiological, clinical, paraclinical, etiological, therapeutic characteristics, and outcomes of AF at Hassan II Regional Hospital in Agadir, Morocco and to compare findings with the available literature.

Methods

Our study consisted of a cross-sectional, retrospective, observational study including 111 patients hospitalized for AF in the Cardiology Department of Hassan II Regional Hospital Center (Agadir) over two years (June 1, 2021, to June 1, 2023). Descriptive analyses were performed using the Excel program.

Results

Our cohort included a total of 111 patients, with a male-to-female ratio of 0.94. The mean age was 63.2 years old. Diabetes and hypertension were the most prevalent risk factors (each 29.7%), and pre-existing heart disease was present in 63%, predominantly ischemic heart disease. Dyspnea was the leading presentation (81.1%). On admission, the mean heart rate was 117 beats per minute (bpm), and clinical signs of heart failure were observed in 71.1%. Electrocardiography showed left bundle branch block in 25.2% and signs of left ventricular hypertrophy in 27%; and chest imaging showed cardiomegaly in 59.5%. Transthoracic echocardiography revealed left atrial dilation in 71.2%, impaired left ventricular systolic function in 55%, and severe mitral stenosis in 17.1%. AF was valvular in 22.5%, while ischemic heart disease was the leading etiology (28.8%). A permanent AF pattern predominated (75.7%). Anticoagulation, guided by thromboembolic risk, was initiated in 90.1% of patients, with vitamin K antagonists (VKAs) used in 62.1%. Rhythm control was chosen in 18.9% versus rate control in 81.1%. The mean hospital stay was 10.3 days, and outcomes were favorable in 70.2%.

Conclusion

AF at Hassan II Regional Hospital in Agadir, Morocco, is characterized by a high comorbidity burden, frequent heart failure at presentation, substantial structural heart disease, and predominance of permanent AF. Anticoagulation was instituted in most patients, with rate control as the primary strategy. These findings underscore the need to strengthen early detection and optimize evidence-based management to prevent severe complications.

## Introduction

Atrial fibrillation (AF) is one of the most common cardiac arrhythmias encountered in clinical practice. It is also a leading cause of ischemic cerebral events [1]. AF is a supraventricular tachyarrhythmia characterized by uncoordinated atrial activation and ineffective atrial contraction. Electrocardiographic features include irregular R-R intervals, absence of distinct P waves, and irregular atrial activity [2]. Its pathophysiology is explained by chaotic electrical activation marked by multiple micro-reentry circuits. This condition leads to hemodynamic inefficiency, thereby increasing the risk of thrombus formation and embolic events [3].

Atrial fibrillation may present with paroxysmal or permanent clinical symptoms, or it may be completely silent and discovered incidentally, underscoring the crucial role of the electrocardiogram (ECG) to confirm the diagnosis [4]. Atrial fibrillation is generally the electrical expression of an underlying cardiac disorder. The classification of atrial fibrillation is based on its clinical and temporal characteristics and includes paroxysmal, persistent, long-standing persistent, and permanent forms [4]. Therapeutic management rests on three pillars: anticoagulation, heart rate control, and rhythm control [3]. Atrial fibrillation is associated with an increased risk of thromboembolic complications [1] and heart failure [5]. The mortality rate in patients with atrial fibrillation is approximately twice that of patients with a normal heart rhythm [3].

Available data on atrial fibrillation in Morocco remain limited and insufficient. At present, most information comes from studies conducted in university hospital centers in different regions of the kingdom. This concentration of data prevents a comprehensive and accurate assessment of the national prevalence and characteristics of AF, highlighting the need for broader and more diverse research. Since it began operating in 2019, the Cardiology Department of the Souss Massa University Hospital, initially based at the Hassan II Regional Hospital Center in Agadir, has received AF patients from multiple regions, offering an opportunity to better explore this cardiac arrhythmia at the regional level.

Our objective is to study and evaluate the epidemiological, clinical, paraclinical, etiological, and therapeutic characteristics and outcomes of AF and to compare our findings with the literature, thereby contributing to knowledge at the national and international levels. Although our study covers the period from June 1, 2021, to June 1, 2023, we used the 2024 European Society of Cardiology (ESC) recommendations, the most recent available at the time of writing.

## Materials and methods

Study design and setting

Our study consisted of a cross-sectional, retrospective, observational study in the Cardiology Department of Hassan II Regional Hospital Center of Agadir, Morocco, over two years (from June 1, 2021, to June 1, 2023). Sampling was carried out exhaustively, including all hospitalized patients who met the inclusion criteria.

Patient population

Patient recruitment was based on hospital department records and discharge letters. A total of 111 patients were included during this period.

Inclusion criteria

**Figure 1:**
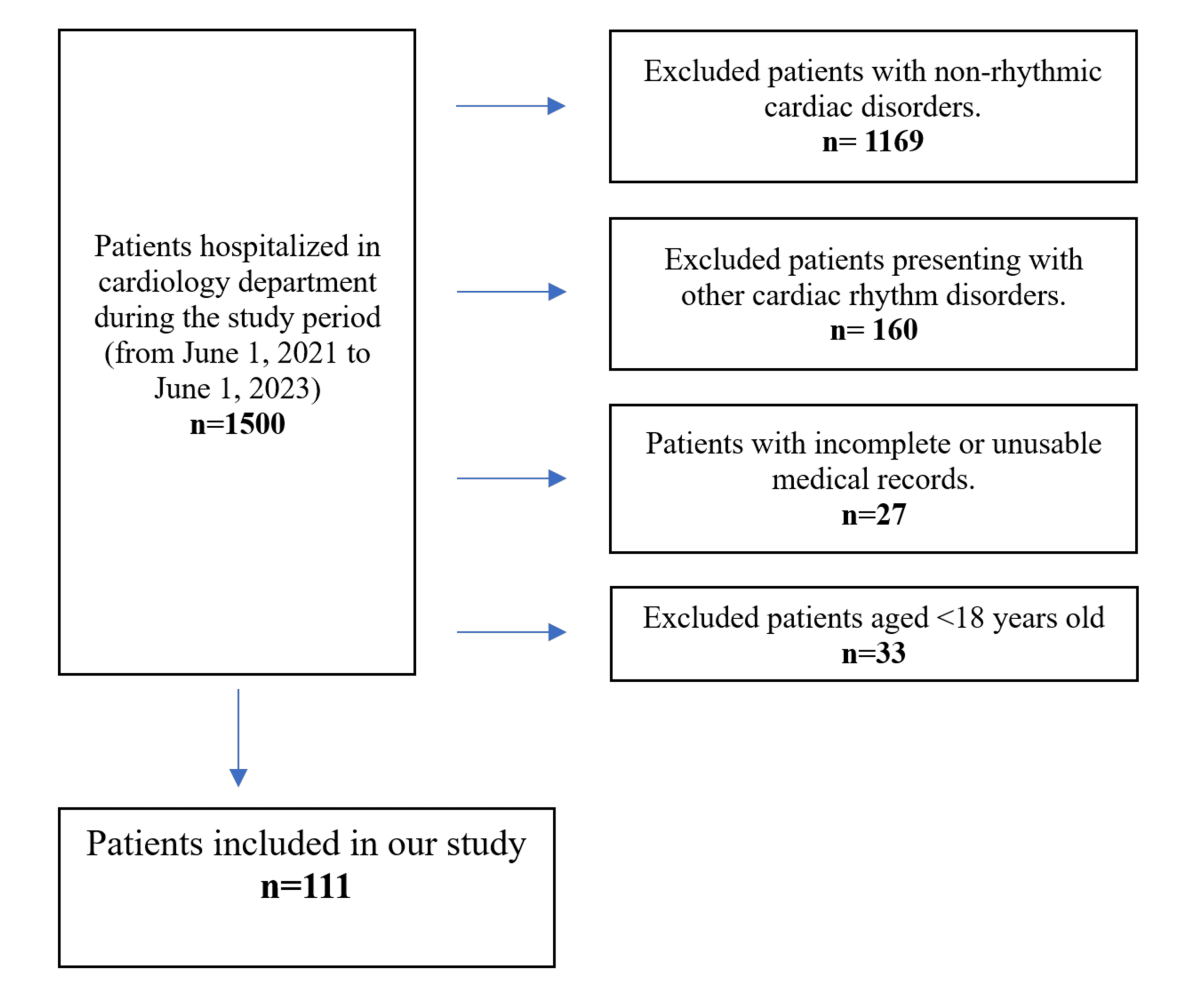
Cohort Flowchart illustrating the inclusion and exclusion criteria in our study.

Eligible participants were adults (≥18 years) admitted to the cardiology department during the study period with atrial fibrillation (AF), either hospitalized primarily for AF or identified with AF on a resting electrocardiogram (ECG) while hospitalized for another condition (Figure 1).

Exclusion criteria

Participants were excluded if they were younger than 18 years, presented with cardiac rhythm disorders other than atrial fibrillation, or had incomplete or unusable medical records or discharge summaries (Figure 1).

Data collection

Data collection was carried out retrospectively using patients’ medical records in both paper and electronic formats. A pre-established data collection sheet for all patients included demographic data; assessed risk factors for atrial fibrillation; documented cardiac and non-cardiac medical histories; recorded the circumstances of discovery; and captured clinical, biological, electrocardiographic, and echocardiographic data. We also reviewed standard chest X-ray findings, determined etiologies, classified atrial fibrillation, calculated CHA₂DS₂-VASc [6] and HAS-BLED [7]scores, detailed therapeutic management, noted length of hospital stay, and described outcomes and complications.

Statistical analysis

The data were analyzed using JAMOVI software version 2.3.28 and entered into Microsoft Excel 2016.

## Results

Patient characteristics

The mean age in our sample was 63.2 ± 15.3 years, ranging from 18 to 110 years. The most affected age group was 61 to 72 years, representing 28.8% (n=32). There was an almost equal gender distribution, with a male-to-female ratio of 0.94.

Past Medical History and Cardiovascular Risk Factors

Diabetes was found in 29.7% (n=33) of patients. Treatment distribution was as follows: 57.6% (n=19) on oral antidiabetics with lifestyle measures, 33.3% (n=11) on insulin therapy with lifestyle measures, 3% (n=1) on both oral agents and insulin, and 6.1% (n=2) untreated. Only 9.1% (n=3) of diabetic patients were well monitored, while 78.8% (n=26) were poorly followed, and 12.1% (n=4) had no follow-up. A degenerative diabetes assessment was performed in only 9.1% (n=3) of diabetic patients, revealing diabetic retinopathy and nephropathy in two patients. Hypertension affected 29.7% (n=33) of patients. Only 69.7% (n=23) received antihypertensive treatment, while 30.3% (n=10) were untreated. Treatment regimens included monotherapy in 65.2% (n=15), dual therapy in 30.4% (n=7), and triple therapy in 4.3% (n=1) of cases. Smoking was the third most frequent cardiovascular risk factor after diabetes and hypertension, found in 27% (n=30) of patients. 12 (10.8%) patients were former smokers. Dyslipidemia was reported in 8.1% (n=9) of patients. Two were managed by lifestyle measures only, and none received lipid-lowering medication. Other risk factors included alcohol consumption, reported in three (2.7%) patients. Obesity (Body Mass Index “BMI” >30 kg/m²) was found in 30 (27%) patients, and 21 were identified as sedentary. Chronic kidney disease was present in 1.8% (n=2), and chronic obstructive pulmonary disease (COPD) in 8.1% (n=9) of patients.

Heart Disease History

In our series, 63% (n=70) of patients were known to have an underlying heart disease at admission. Non-revascularizable coronary disease was the most frequent, affecting 11 (9.9%) patients. The second most common condition was severe mitral stenosis, observed in 8 (7.2%) patients. In 24 cases, the etiology of the cardiopathy was not specified. In our study, 6.3% (n=7) of patients had previously undergone cardiac surgery. These included four cases of combined mechanical mitro-aortic valve replacement with tricuspid valve repair, two cases of isolated mechanical mitral valve replacement, and one case of percutaneous mitral commissurotomy (Table 1).

**Table 1:** Prevalence of known heart disease in the study population.

Type of Heart Disease	Percentage	Number of patients
Undocumented heart disease	21.60%	24
Non-revascularizable coronary disease	9.90%	11
Severe mitral stenosis (tight MS)	7.20%	8
Cardiac surgery	6.30%	7
Aortic and/or mitral valvulopathy (without severe mitral stenosis)	6.30%	7
Dilated cardiomyopathy	4.50%	5
Ischemic heart disease (revascularizable)	2.70%	3
Hypertensive heart disease	1.80%	2
Hypertrophic cardiomyopathy	0.90%	1
Ebstein’s anomaly	0.90%	1
Unoperated atrial septal defect	0.90%	1

In our series, 28.8% (n=32) of patients reported other pathological histories. The most frequently noted conditions were thyroid disorders (3.6%, n=4) and cerebrovascular accident (3.6%, n=4). Other less common conditions, each representing 1.8% (n=2) or fewer, included epilepsy, bladder tumor, lower limb fracture, gout, rheumatoid arthritis, bone and joint infection, ortal vein thrombosis, cataract, gastric tumor, brain tumor, and breast tumor.

Presenting Symptoms

**Figure 2:**
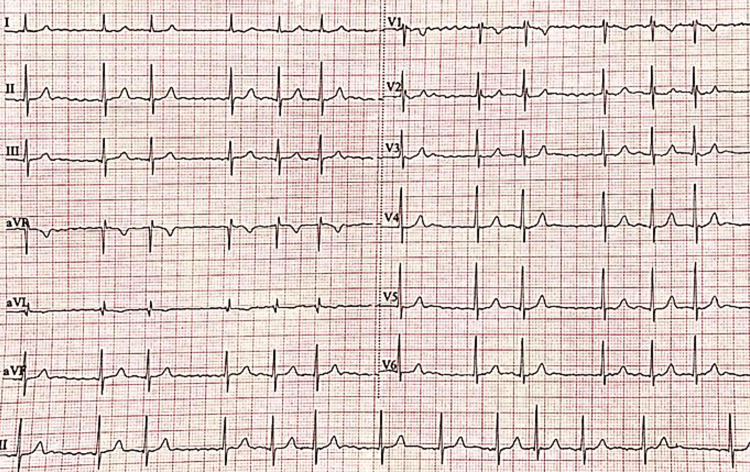
Typical electrocardiographic appearance of atrial fibrillation: Absence of P waves. ECG of a 37-year-old woman with no significant medical history who presented to the emergency department with recurrent palpitations.

Dyspnea was the most frequently reported reason for consultation in cases of AF, with a frequency of 81.1% (n=90). NYHA class IV in 39 (35.1%) patients and NYHA class III in 20 (18%) patients. Palpitations were reported by 19.8% (n=22) of patients. Other less common presenting symptoms included chest pain in 13.5% (n=15) of cases, lower limb swelling in 13.5% (n=15), syncope in 0.9% (n=1), neurological signs in 1.8% (n=2), bleeding in 4.5% (n=5), and fever in 0.9% (n=1). On ECG, the cardiac rhythm was irregular in 94.6% (n=105) of patients (Figure 2). Additionally, left ventricular hypertrophy (LVH) was identified in 30 (27%) patients, while right ventricular hypertrophy (RVH) was present in 4 (3.6%) patients. A wide QRS complex was observed in 47 (42.3%) patients, with left bundle branch block being the most frequent abnormality. Repolarization disorders were found in 74 (66.7%) patients, and microvoltage was noted in 4 (3.6%) cases (Table 2).

**Table 2:** Abnormal findings on cardiac examination in the study population.

Electrocardiographic abnormality	Percentage	Number of patients
Left ventricular hypertrophy (electrical)	27%	30
Right ventricular hypertrophy (electrical)	3.60%	4
Wide QRS complex	-	-
Left bundle branch block	25.20%	28
Right bundle branch block	13.50%	15
Nonspecific intraventricular block	3.60%	4
Repolarization abnormalities	-	-
Systematized	22.50%	25
Non-systematized	44.10%	49
Microvoltage	3.60%	4

Physical Examination

Almost all our patients were neurologically stable. The mean systolic blood pressure (SBP) was 125 mmHg, ranging from 80 mmHg to 190 mmHg. The mean diastolic blood pressure (DBP) was 74.2 mmHg, ranging from 40 mmHg to 120 mmHg. The mean heart rate (HR) was 117 bpm, ranging from 54 bpm to 220 bpm. The mean oxygen saturation was 95.8%, with extremes between 79% and 99%. Only one patient (0.9%) was febrile upon admission. Nine (8.1%) patients showed signs of peripheral hypoperfusion at admission.

On cardiac auscultation, 108 (97.3%) patients presented irregular heart sounds. A cardiac murmur was detected in 44.1% (n=49) of cases. In 89,8% (n=44) of those patients, it was audible at the mitral valve, with a maximum intensity of 5/6 in 17 of them. At cardiovascular examination, 71.1% (n=79) of patients presented with clinical symptoms of heart failure: 47.7% (n=53) patients showed signs of left-sided heart failure, and 49.5% (n=55) showed signs of right-sided heart failure. Twenty-nine patients presented with global heart failure, representing 26.1% of the total.

Laboratory Assessment

Anemia was found in 36% (n=40) of patients. The mean hemoglobin level was 12.5 g/dL, with values ranging from 5.6 g/dL to 17.4 g/dL. Blood count showed hyperleukocytosis in 41.4% (n=46) of patients, 56.8% (n=63) had a normal white blood cell count, and two patients presented leukopenia. Neutrophil levels were elevated in 39.6% (n=44) of patients and normal in 60.4% (n=67). Platelet counts were within normal limits in 94.6% (n=105) of cases, while 5.4% (n=6) of patients had thrombocytopenia. C-reactive protein (CRP) levels were normal (<6 mg/L) in 24.3% (n=27) of patients. Blood electrolytes showed hyperkaliemia in four (3.6%) patients, while four others had hypokalaemia. The mean serum potassium level was 4.37 mmol/L. Hyponatraemia was noted in 31.5% (n=35) of patients, whereas 1.8% (n=2) presented with hypernatraemia. The mean serum sodium concentration was 135 mmol/L. Renal impairment with elevated urea levels was observed in 20.7% (n=23) of patients. The mean serum creatinine level was 13 mg/L, while the mean urea level was 0.66 g/L. Liver function tests revealed hepatic cytolysis in 12.6% (n=14) of patients and cholestasis in 4.5% (n=5). Prothrombin time (PT) was decreased in 28.8% (n=32) of cases, and activated partial thromboplastin time (aPTT) was prolonged in 1.8% (n=2) of patients. High-sensitivity troponin (hs-Tn) assays were performed in 19.8% (n=22) of patients and found to be elevated in 10.8% (n=12) of them, representing 54.5% (n=12) of those tested and 10.8% (n=12) of the total study population. NT-proBNP testing was requested in 12.21% (n=11) patients and was positive in 11.1% (n=10), corresponding to 90.9% (n=10) of those tested and 9% (n=10) of the total study population (Table 3).

**Table 3:** Summary of main laboratory findings in the study population. NT-proBNP: N-terminal pro-B-type natriuretic peptide; hs-Tn: High-Sensitivity Troponin; PT: Prothrombin time; CRP: C-reactive protein; Hb: Hemoglobin

Parameter	Category	n (% of total patients)	Additional details
Hemoglobin	Anemia	40 (36.0%)	Mean Hb: 12.5 g/dL (range 5.6-17.4 g/dL)
White blood cell count	Hyperleukocytosis	46 (41.4%)	
	Normal	63 (56.8%)	
	Leukopenia	2 (1.8%)	
Neutrophils	Elevated (neutrophilia)	44 (39.6%)	
	Normal	67 (60.4%)	
Platelets	Normal count	105 (94.6%)	
	Thrombocytopenia	6 (5.4%)	
CRP	Normal (< 6 mg/L)	27 (24.3%)	
	Elevated (≥ 6 mg/L)	84 (75.7%)	
Potassium	Hyperkalaemia	4 (3.6%)	Mean serum K⁺: 4.37 mmol/L
	Hypokalaemia	4 (3.6%)	
Sodium	Hyponatraemia	35 (31.5%)	Mean serum Na⁺: 135 mmol/L
	Hypernatraemia	2 (1.8%)	
Renal function	Renal impairment with elevated urea	23 (20.7%)	
	-	-	Mean creatinine: 13 mg/L, mean urea: 0.66 g/L
Liver function	Hepatic cytolysis	14 (12.6%)	
	Cholestasis	5 (4.5%)	
Coagulation tests	Decreased PT	32 (28.8%)	
	Prolonged activated partial thromboplastin time	2 (1.8%)	
High-sensitivity troponin	Patients tested	22 (19.8%)	hs-Tn assay performed in 22 patients
	Elevated hs-Tn	12 (10.8%)	54.5% of those tested
NT-proBNP	Patients tested	11 (9.9%)	NT-proBNP assay requested in 11 patients
	Elevated NT-proBNP	10 (9.0%)	90.9% of those tested

Imaging techniques

**Figure 3:**
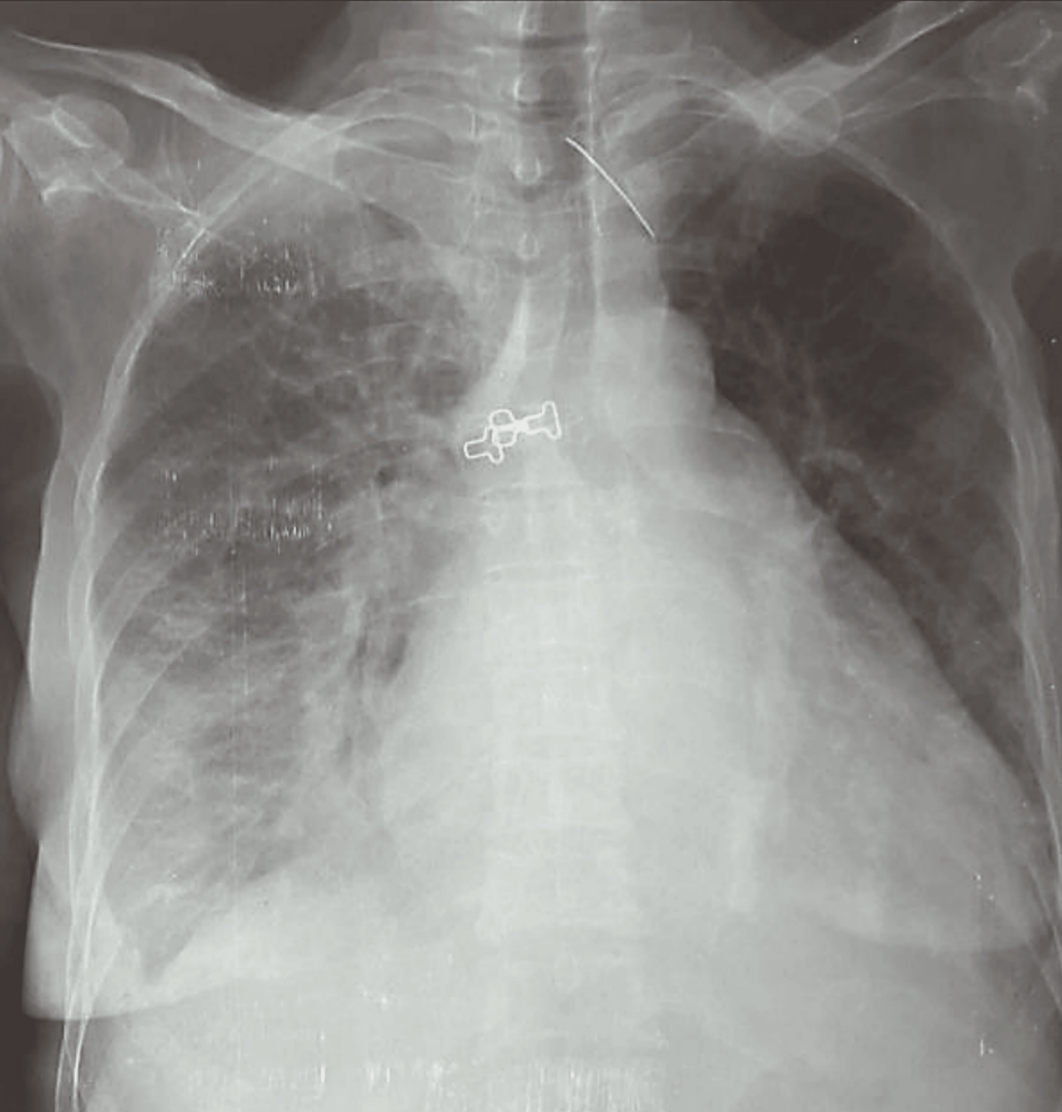
Chest X-ray showing a mitral configuration of the cardiac silhouette: cardiomegaly with a supradiaphragmatic apex, convexity of the mid-left cardiac border, and a double contour (double density) of the right lower cardiac border. Chest X-ray of a 45-year-old woman presenting with shortness of breath and palpitations.

Chest X-ray revealed cardiomegaly in 59.4% (n=66) patients, with a “mitral silhouette” appearance in 10.81% (n=12) of cases (Figure 3). Other anomalies included bilateral alveolar syndrome, hilar congestion, and pleural effusion, the latter was found in 32.4% (n=36) of patients, which was unilateral in 21.6% (n=24) of cases, and minimal in 69.4% (n=77) of cases.

Heart Chambers

Transthoracic echocardiography (TTE) revealed left ventricular (LV) dilation in 29.7% (n=33) of patients. The mean left ventricular end-diastolic diameter (LVEDD) was 52.1 mm, ranging from 37 mm to 69 mm. The mean left ventricular end-systolic diameter (LVESD) was 36.6 mm, with values between 20 mm and 67 mm. LVH was observed in 16.2% (n=18) of patients: concentric in 15.3% (n=17) and eccentric in 0.9% (n=1) of cases.

Left ventricular ejection fraction (LVEF) was preserved (≥50% ejection fraction) in 45% (n=50) of patients, moderately reduced (40-49%) in 17.2% (n=19), and reduced (<40%) in 37.8% (n=42). Segmental wall motion abnormalities were observed in 46.8% (n=52) of patients, with akinesia in 23.4% (n=26), hypokinesia in 22.5% (n=25), and dyskinesia in 0.9% (n=1). Global wall motion abnormalities were found in 25.2% (n=28) of patients.

Heart Contraction

Left ventricular diastolic dysfunction was present in 26.1% (n=29) of patients. Regarding the left atrium (LA), dilation was observed in 79 patients, accounting for 71.2% of the cohort. This was defined as a left atrial area (LAA) greater than 20 cm², with a mean value of 36.6 cm² (ranging from 16 to 65 cm²). An intra-atrial thrombus was detected in 1.8% (n=2) of patients, while spontaneous echo contrast was noted in 3.6% (n=4) of patients. Right ventricular (RV) evaluation showed normal dimensions in 48.6% (n=54) of cases, dilation in 48.6% (n=54), and hypertrophy in 2.7% (n=3). Right ventricular longitudinal systolic function was impaired in 53.2% (59 cases) of patients, with a mean tricuspid annular plane systolic excursion (TAPSE) of 16 (ranging from 8 to 29 mm). Mean systolic pulmonary arterial pressure (sPAP) was 32 mmHg, varying between 19 and 92 mmHg. Finally, the right atrium (RA) was dilated in 63.1% (n=70) of patients, whereas 36.9% (n=41) had a normal size. Dilation was defined as a right atrial area (RAA) greater than 18 cm².

Valve Function

Echocardiographic evaluation of the mitral valve showed a well-functioning mechanical prosthesis in 5.4% (n=6) of patients. Mitral regurgitation was present in 61.3% (n=68) of patients, of which 34.2% (n=38) had grade II or higher regurgitation. Mitral stenosis was observed in 27% (n=30) of patients and was classified as severe in 17.1% (n=19) of cases. Assessment of the aortic valve revealed a well-functioning mechanical prosthesis in 3.6% (n=4) of patients. Aortic regurgitation was found in 31.5% (n=35) of patients, while aortic stenosis was noted in 10.8% (n=12). Evaluation of the tricuspid valve demonstrated prior annuloplasty in 3.6% (n=4) of patients. Tricuspid regurgitation was observed in 36% (n=40) of patients, while no cases of tricuspid stenosis were identified. The pulmonary valve appeared thin and normal in all patients, with no evidence of regurgitation or stenosis.

Pericardium

Pericardial effusion was identified in 19.8% (n=22) of patients. The effusion was mild in 17.1% (n=19) and moderate in 2.7% (n=3) of cases.

Types of AF

In our study, permanent atrial fibrillation was the most common form, accounting for 75.7% (n=84) of cases. Persistent and long-standing persistent AF ranked second with 15.3% (n=17), while paroxysmal AF accounted for 9% (n=10).

Etiological evaluation of AF

In our series, 22.5% (n=25) of patients had valvular AF, including those with moderate-to-severe or severe mitral stenosis, which was found in 17.1% (n=19) of patients. Mechanical prostheses accounted for 5.4% (n=6) of cases. Non-valvular causes were identified in 60.3% (n=67) of patients. These included chronic coronary syndrome in 24.3% (n=27) of patients, followed by dilated cardiomyopathy in 10.8% (n=12). Aortic and/or mitral valve disease (excluding severe mitral stenosis) represented 5.4% (n=6) of cases. Hypertensive cardiomyopathy, acute coronary syndrome, and restrictive cardiomyopathy were each found in 4.5% (n=5) of patients, while hypertrophic cardiomyopathy was present in 1.8% (n=2). Other rare causes were also identified, each representing 0.9% (n=1) of cases: chronic constrictive pericarditis, infective endocarditis, unoperated atrial septal defect, Ebstein’s anomaly, and chronic pulmonary heart disease. Other non-cardiac etiologies accounted for 8.2% (n=9) of all causes. The most frequent among them was hyperthyroidism, found in 5.4% (n=6) of patients. Pulmonary embolism was observed in 1.8% (n=2) of cases, while infectious bronchopneumonia represented 0.9% (n=1). Finally, no etiology was identified in 9% (n=10) of patients.

Thromboembolic and bleeding risk assessment

All patients without valvular atrial fibrillation, 77.5% (n=86), underwent thromboembolic risk assessment using the CHA₂DS₂-VASc score to determine the indication for anticoagulation. In our cohort, 3.6% (n=4) of patients (2 men and 2 women) had a score of 0, while 15.3% (n=17) patients (10 men and 7 women) had a score of 1. 65 patients had a score of 2 or higher, including 35 men and 30 women, 4 of whom had a score exactly equal to 2. The HAS-BLED score was calculated in patients with non-valvular atrial fibrillation (77.5%, n=86) to assess bleeding risk. Results showed that 59.4% (n=66) of patients had a score strictly below 3, whereas 18% (n=20) of patients had a score of 3 or higher.

Therapeutic management

In our study, the vast majority of patients with AF (90.1%, n=100) were given anticoagulation therapy. All patients with valvular AF received anticoagulation. Among those with non-valvular AF, anticoagulation was indicated in 90.7% (n=78) of cases (Table 4).

**Table 4:** Rate of anticoagulation use among patients with valvular AF and non-valvular AF (by CHA₂DS₂-VASc score). AF: Atrial Fibrillation

Category	Percentage	Number of patients
Score ≥ 2 in men and ≥ 3 in women	93.40%	104
Score = 1 in men and = 2 in women	92.80%	103
Score = 0 in men and 0–1 in women	54.50%	60
Valvular FA	100%	25

In our series, the anticoagulant treatments administered included: vitamin K antagonists (VKAs) in 62.1% (n=69) of patients; low-molecular-weight heparin (LMWH) in 3 patients, with no use of heparin analogues such as fondaparinux; and direct oral anticoagulants (DOACs) in 25.2% (n=28) of patients, 24.3% (n=27) receiving rivaroxaban and 0.9% (n=1) receiving apixaban. No patient was treated with dabigatran or edoxaban. Finally, 9.9% (n=11) of patients did not receive any form of anticoagulation.

Rhythm Control Strategy

A rhythm control approach was used in 18.9% (n=21) of patients. Electrical cardioversion was performed in 8 patients. Electrical shock was indicated in 5 patients due to poor tolerance of AF. Pharmacological cardioversion was chosen in 80.9% (n=17) of rhythm-control cases. Amiodarone was used in all patients. Four patients received pharmacological cardioversion followed by electrical cardioversion. The success rate of cardioversion was 66.7% (n=74). A sinus rhythm maintenance strategy after cardioversion was implemented in 76.2% (n=16) of patients who had undergone rhythm reduction, and amiodarone was used in all these cases. None of the patients in our study underwent atrial fibrillation ablation.

Rate Control Strategy

A rate control approach was adopted in 81.1% (n=90) of patients. In 5 cases, it was considered as a second-line option after failed rhythm control. Beta-blockers were the most commonly used medications, prescribed in 79.2% (n = 88) of patients. Among these, bisoprolol was the most frequently prescribed beta-blocker, used in 68.5% (n = 76) of patients, including those receiving bisoprolol alone (n = 60) and those receiving a combination of bisoprolol and digoxin (n = 16). Carvedilol was prescribed in 7.2% (n = 8) of patients, including those receiving carvedilol alone (n = 5) and those receiving a combination of carvedilol and digoxin (n = 3), and nebivolol in 1.8% (n = 2) of patients. Monotherapy was prescribed in 68.4% (n=76) patients, while combined therapy was used in 17.1% (n=19). The most common combination was bisoprolol with digoxin (Table 5).

**Table 5:** Different drugs and combinations used to control the heart rate in our patients.

Medication	Percentage	Number of patients
Bisoprolol + Digoxin	14.40%	16
Nebivolol	1.80%	2
Carvedilol + Digoxin	2.70%	3
Calcium channel blocker	0.90%	1
Carvedilol	4.50%	5
Propranolol	1.80%	2
Digoxin	5.40%	6
Bisoprolol	54%	60

Heart failure treatment:

Loop diuretics, mainly furosemide, were prescribed in 98 patients. Beta-blockers were used in 79.2% (n=88) of patients, and spironolactone in 42.3% (n=47). Angiotensin-converting enzyme inhibitors (ACEIs) were prescribed in 38.7% (n=43) of patients: ramipril in 25.2% (n=28), perindopril in 9.9% (n=11), and captopril in 3.6% (n=4). Angiotensin receptor-neprilysin inhibitors (ARNIs) were used in 2.7% (n=3) of patients. Angiotensin II receptor blockers (ARBs), represented by valsartan, were prescribed in 5 patients. Sodium-glucose co-transporter 2 (SGLT2) inhibitors were prescribed in 4.5% (n=5) of patients, dapagliflozin in 3.6% (n=4) and empagliflozin in 0.9% (n=1) (Table 6).

**Table 6:** Different drugs used to treat heart failure in our patients. ACE: Angiotensin-converting enzyme inhibitors; SGLT2: Sodium-glucose co-transporter 2

Medication	Percentage	Number of patients
Loop diuretics	88.20%	98
Spironolactone	42.30%	47
ARNI (angiotensin receptor–neprilysin inhibitor)	2.70%	3

Regarding antiplatelet therapy for ischemic heart disease, 39.6% (n=44) of patients were on antiplatelet treatment. Among them, 38 (34.2%) patients received aspirin monotherapy, one patient was treated with clopidogrel alone, and five (4.5%) patients were on dual antiplatelet therapy combining aspirin and clopidogrel. In addition, statins were prescribed in 48.6% (n=54) of patients. None (8.1%) of the patients in our series underwent coronary revascularization procedures (Table 7).

**Table 7:** Different antiplatelet drugs used in our patients.

Antiplatelet therapy	Percentage	Number of patients
Aspirin	34.20%	38
Clopidogrel	0.90%	1
Aspirin + Clopidogrel	4.50%	5

Sixteen (14.4%) patients in our study received antibiotic therapy, representing 14.4% (n=16) of the cohort. Six (5.4%) patients were treated with synthetic antithyroid medications. The mean duration of hospitalisation was 10.3 ± 5.8 days, ranging from 1 to 28 days. Clinical evolution was favourable in 78 (70.2%) patients. The main complications observed were thromboembolic events of the ischemic stroke type in 6 (5.4%) patients, and haemorrhagic complications in another six (5.4%) patients (including 3 haemorrhagic strokes, two digestive haemorrhages, and one urogenital haemorrhage). Conduction disorders were observed in three (2.7%) patients (two cases of high-degree atrioventricular block and one case of bradyarrhythmia with AF). De novo heart failure secondary to atrial fibrillation occurred in 16 (14.4%) patients. Finally, two (1.8%) patients died in a context of cardiogenic shock (Table 8).

**Table 8:** Outcome and complications observed in our patients. AF: Atrial Fibrillation

Outcome / Complication	Percentage	Number of patients
Favorable course	70.20%	78
New-onset heart failure related to AF	14.40%	16
Hemorrhagic complications	5.40%	6
Thromboembolic complications	5.40%	6
Conduction disorders	2.70%	3
Death	1.80%	2

## Discussion

Definition

According to the 2024 recommendations of the European Society of Cardiology (ESC), AF is defined as a supraventricular cardiac arrhythmia characterized by uncoordinated atrial electrical activation, leading to the loss of effective atrial contraction. On ECG, it presents with irregular R-R intervals, absence of distinct repeating P waves, and irregular ventricular activations [8]. AF is a supraventricular arrhythmia marked by rapid and completely irregular atrial depolarization, typically arising from ectopic foci in the pulmonary veins or atria with abnormal automaticity, and/or multiple micro-reentry circuits due to atrial fibrosis [9]. Micro-reentry is an electrophysiological phenomenon in which a small, abnormal electrical circuit forms within cardiac tissue, typically around damaged areas or abnormal conducting fibers, generating repetitive, self-sustaining impulses that disrupt normal rhythm and contribute to arrhythmias such as atrial fibrillation. Unlike macro-reentry, which involves larger circuits, micro-reentry is smaller and often harder to detect and treat [10]. Several factors promote micro-reentry in atrial fibrillation. Fibrosis remodels atrial structure and electrophysiology, and oxidative stress drives electrical and structural remodeling that facilitates reentrant circuits. Inflammation supports both the initiation and maintenance of AF by inducing structural and electrophysiological changes. Other AF risk factors include aging, heavy alcohol consumption, and physical inactivity [11].

Epidemiology

AF is the most common cardiac arrhythmia; the global prevalence of AF in 2017 was estimated at 37.574 million cases [12]. Risk factors include population aging, valvular heart disease, diabetes mellitus, hypertension, obesity, as well as metabolic syndrome, sleep apnea, and inflammation. Genetic factors and lifestyle factors such as alcohol consumption and physical activity may also play a role [13]. In our study, the mean patient age was 63.2 years, findings consistent with the Global Health Data Exchange, regarded as one of the most comprehensive health-data catalogues. Nationally, the mean age varies: in Rabat, Bouzelmat et al. (2015) reported 57 ± 14 years [14]; in Casablanca, Cheikh Khalifa University Hospital study (2019) reported a higher mean age of 70 years old [15]. In Framingham, incidence was 1.6/1,000 person-years in women vs 3.8/1,000 in men (male predominance) [16-18]. Sex distribution can vary; Thiès reported female predominance (sex ratio 0.7) [19]. Outcomes differ by sex: BiomarCaRE found a higher adjusted mortality risk in women [18]. In our cohort, sex distribution was nearly equal (sex ratio 0.94).

In our cohort, AF prevalence was 35.1% (111/316 admissions), closely matching CHU Hassan II Fez (36%) [20] but exceeding Avicenne Military Hospital, Marrakech (10.8%). Internationally, AF prevalence varies widely, 8-40% in a European multicenter analysis depending on diagnostic approach [21], about 20% among cardiovascular inpatients in Australia [22], up to 40% in U.S. cardiology specialty hospitals [23], and 12-35% across other European reports [24].

Classifications of atrial fibrillation

The European Society of Cardiology (ESC) distinguishes five stages [8,17], the first diagnosed AF, a previously undiagnosed episode regardless of duration or symptom burden. Paroxysmal AF terminates within seven days of onset, typically within 48 hours. Persistent AF lasts >7 days and terminates spontaneously or after cardioversion. Long-standing persistent AF, continuous >12 months, with a rhythm-control strategy considered. Permanent AF, accepted by clinician and patient with no attempt to restore sinus rhythm [8,17]. Clinical-concept classification (ESC 2024). Forms include clinical AF, symptomatic or asymptomatic AF diagnosed on surface ECG; the minimum duration for diagnosis remains unclear [9]. Subclinical AF, asymptomatic AF episodes detected by implantable cardiac devices predict subsequent clinical AF. AF burden, the total duration of AF over a monitoring period, is often expressed as a percentage. Trigger-induced AF, secondary to a potentially reversible factor [8]. Self-terminating AF, paroxysmal AF that ends spontaneously without cardioversion. Non-self-terminating AF does not end spontaneously or requires cardioversion [25]. Valvular AF is defined by the presence of moderate-to-severe or severe mitral stenosis or a mechanical valve prosthesis. Non-valvular AF encompasses all other etiologies; this distinction remains important for thromboembolic risk assessment and anticoagulation decisions [26].

Clinical symptoms

AF has a highly variable clinical expression, largely influenced by underlying heart disease, most often hypertension or rheumatic valvular disease. Classic symptoms include palpitations, dyspnea, which may significantly impair quality of life, and it can also first manifest through complications. Many patients remain asymptomatic, complicating diagnosis [27]. Symptom assessment should be performed at baseline, after treatment changes, and before/after procedures [8]. Symptom presence or absence does not predict stroke, systemic embolism, or mortality, but symptom burden substantially reduces quality of life [28]. Specific symptoms like palpitations are less frequent than nonspecific ones, such as fatigue, and women tend to be more symptomatic with poorer quality of life [29]. In our cohort, dyspnea was the leading symptom (81.1%), consistent with prior series identifying dyspnea as predominant [20].

Physical examination

On physical examination, first evaluate hemodynamic stability and signs of heart failure. The rhythm is typically irregular, and the ventricular rate is variable. A thorough exam should seek clues to underlying causes (e.g., hypertension, valvular murmurs, pulmonary disease, thyroid dysfunction) and complications, warranting routine neurological and vascular assessment [27]. In our cohort, the mean heart rate was 117 bpm, higher than in the series by L. Ouaha, I. Zouidine, and Y. Dannouni, though still within their reported ranges [19]. Our mean blood pressure was 125/74.2 mmHg, both lower than in the Dannouni study [30].

Electrocardiogram

The ECG establishes the diagnosis in the vast majority of cases, showing atrial activity with no P waves but irregular fibrillatory waves that give a tremulous, polymorphic baseline. Ventricular activity features irregular RR intervals, QRS complexes are usually narrow but may be wide with bundle-branch block, pre-excitation, or 2nd degree atrioventricular (AV) block with infra-nodal escape. Rates rarely exceed 180 bpm except with pre-excitation. Associated findings can include secondary repolarization changes, signs of underlying cardiopathy. Practically, any irregular narrow-complex tachycardia should be considered AF until proven otherwise [31]. External Holter ECG is used diagnostically to detect intermittent AF in patients with palpitations or ischemic stroke when a standard ECG is normal [27]. Exercise ECG is useful when AF is exercise-induced [27]. In our cohort, persistent and permanent atrial fibrillation were the predominant types. An abnormal ECG was present at admission in 105 patients, while 6 had a normal ECG at first evaluation, findings consistent with paroxysmal AF.

In our cohort, ECG abnormalities in AF were frequent: LVH 27%, similar to Ouaha 27.3% [20], higher than Zouidine 18% [32], and between Dannouni 22% [30] and Affangla 32.8%; LBBB 25.2%, exceeding Zouidine 17% [32] and Dannouni 9% [30]; RBBB 13.5%, between Zouidine 17% [32] and Dannouni 8% [30]. Collectively, these findings align with literature showing common structural/ischemic associations in AF while highlighting higher conduction and repolarization abnormalities in our series.

Laboratory examination

Thyroid panel and serum electrolytes are recommended in all patients presenting with de novo AF according to European and American society guidelines [16,17]. Renal panel with eGFR calculation, liver panel, complete blood count, and coagulation studies constitute the pre-treatment work-up before initiating anticoagulation [16,17].

Imaging findings

Chest radiography allows the detection of structural cardiac abnormalities such as left atrial enlargement and signs of pulmonary congestion [33]. Transthoracic echocardiography (TTE), including two-dimensional imaging and comprehensive Doppler valve assessment, is recommended for all patients with AF. It helps identify underlying heart diseases predisposing to AF, notably impaired left ventricular systolic function due to ischemic or dilated cardiomyopathy, left ventricular hypertrophy, valvular heart disease or pericardial disease. TTE provides a rapid, non-invasive evaluation of cardiac structures, notably left atrial and left ventricular dimensions, as well as left ventricular systolic and diastolic function [34]. Transoesophageal echocardiography (TEE) enables high-resolution examination of posterior cardiac structures, notably the atria, interatrial septum, and pulmonary veins. More importantly, the atrial appendages can be visualized to detect thrombus. A more precise assessment of valvular lesions, particularly prosthetic dysfunction, is possible with TEE. Indications include peripheral arterial embolism (to search for intra-atrial thrombus), attempting electrical cardioversion without waiting for 3 weeks of effective anticoagulation, before endocavitary ablation of AF, and as a second-line test in a poorly echogenic patient on TTE [34]. In our cohort, LV systolic dysfunction was 55%, lower than I. Zouidine 77% [32] and F. Keita 65.8% [35]. Left-atrial dilation was 71.2%, comparable to D. Affangla 71.42% [19] and between F. Keita 63.2% [35], I. Zouidine 75% [32], and Y. Dannouni 83% [30]. Mitral stenosis was 27%, aligning with Dannouni 25% [30] and Affangla 28% [19]. Other frequent findings in our series were right-atrial dilation 63.1%, right-ventricular dilation 48.6%, and pericardial effusion 19.8%.

Differential diagnoses

The differential diagnosis of typical AF, usually an irregular narrow-QRS tachycardia, includes: atrial flutter with variable conduction (especially coarse “AF-like” flutter lacking a clear isoelectric baseline), focal or multifocal atrial tachycardia, bursts of atrial ectopy (<10 seconds), and electrical artefacts (e.g., tremor in Parkinson’s disease). When AF is rapid with wide QRS complexes (due to bundle-branch block or pre-excitation), the key alternative to consider is ventricular tachycardia [31].

Etiologies

Mitral valve disease is most frequently implicated (mitral stenosis, mitral regurgitation, mitral valve disease), followed by aortic valve disease. Tricuspid valve disease can also cause AF through right atrial dilation. Valve prostheses (mechanical and bioprosthetic) may be complicated by AF, with a very high thromboembolic risk for mechanical valves [9,27]. Cardiomyopathies at risk of developing AF include mainly hypertensive heart disease. All other cardiomyopathies (acute or chronic ischaemic, hypertrophic, restrictive, etc.) can also be responsible for AF. Other cardiac etiologies include post-cardiac surgery, acute pericarditis, myocarditis, and congenital heart disease [9,27]. Extracardiac etiologies include hyperthyroidism, acute lung diseases (infection, pulmonary embolism, etc.), electrolyte disorders (hypokalaemia, hypomagnesaemia), iatrogenic causes (notably direct and indirect beta-sympathomimetics: dobutamine, dopamine, noradrenaline, salbutamol, isoprenaline), acute alcohol intake, vagal AF, and adrenergic AF (triggered by exertion, stress, fever, or pain). Idiopathic AF is a diagnosis of exclusion, occurring in a young individual without risk factors [9,27]. In our study, valvular AF accounted for 22.5%, similar to Y. Dannouni and F. Keita but lower than H. Bouzelmat et al. (60%) [14] and higher than a Dutch tertiary-center cohort where valvular AF was 9.1% among 1,885 patients, severe mitral stenosis caused 17.1% of cases vs 35% in Dannouni [30]. Non-valvular AF comprised 77.5%, chiefly linked to ischemic and dilated cardiomyopathy, aligning with Keita and with Moroccan reports emphasizing ischemic/hypertensive heart disease. Idiopathic AF was 9%, comparable to Dannouni and Keita [30,35].

Therapeutic management

AF care follows the four “CARE” pillars: Comorbidity and risk-factor management; Avoid stroke; Reduce symptoms (rate/rhythm control); and Evaluation with dynamic reassessment [8]. Managing comorbidities by targeting a BP around 120-129/70-79 mmHg, optimise diabetes [8], reduce weight in obesity [8], prescribe exercise for paroxysmal/persistent AF to lower recurrence [36], and curb alcohol to lessen AF burden [8]. For stroke prevention, valvular AF (moderate-severe MS or mechanical valve) needs VKA anticoagulation, in non-valvular AF, use CHA₂DS₂-VA (anticoagulate if ≥2; recommend if =1) and assess bleeding with HAS-BLED (≥3 high risk) [8,27].

Cardioversion, if AF <48 h, proceed without pre-anticoagulation but continue 4 weeks after, if >48h or unknown, give ≥3 weeks before and ≥4 weeks after, or TEE-guided strategy with ≥4 weeks post-procedure [27]; long-term indications are similar across AF types. VKAs are required for MS/mechanical valves; in non-valvular AF, DOACs are preferred for similar efficacy and lower ICH risk [8,27]. Percutaneous Left Atrial Appendage may be considered when anticoagulation is contraindicated [8,17]. Rhythm control is prioritised for symptomatic patients, younger age, reversible AF, or AF with heart failure. Cardioversion (electrical/pharmacological) is used when poorly tolerated or elective, but rhythm control is less favorable in long-standing persistent AF, prior failed cardioversion, or HF with LV dysfunction [8,27]. Antiarrhythmic drugs reduce recurrences but carry cardiac/extracardiac risks and are mainly for symptomatic/poorly tolerated AF [36]. Catheter ablation is more effective in paroxysmal/persistent AF, with evolving options such as pulsed-field ablation and selected surgical/endoscopic approaches [37,38]. Continuous evaluation and dynamic reassessment ensure treatment remains tailored over time [8].

Complications and prognosis

AF complications include high thromboembolic risk, especially stroke (greatest in valvular AF), heart-failure decompensation, frequent recurrences, atrial fibrosis-related tachy-brady, reversible tachycardia-induced cardiomyopathy, and treatment-related complications (drug-induced conduction blocks, anticoagulation-related bleeding) [27]. Studies show that individuals with AF have higher mortality than those without, independent of demographic and clinical factors: in the Framingham Heart Study (5,209 participants; 40-year follow-up), mortality was 90% higher in women and 50% higher in men who developed AF, a pattern echoed in the Renfrew cohort (15,406 participants) with 1.5 to 2-fold higher mortality in both sexes [39, 40]. In our cohort, outcomes were favorable in 70.2%, slightly higher than Zouidine 66% [32]; thromboembolic complications 5.4% were comparable to Dannouni 6% [30] and Ouaha 5.3% [20]; hemorrhagic complications 5.4% were lower than Zouidine 13% [32] and Dannouni 9% [30]; de novo heart failure 14.4% was far below Ouaha 53.3% [20] but above Dannouni 8% [30]; and mortality 1.8% was below Zouidine 2% [32] and well below Ouaha 4.7% [20].

Limitations of our study

This was a retrospective study with potential selection bias (milder/asymptomatic AF may be missed) and missing or incomplete records, regional generalizability is limited to a single hospital with possible treatment-practice biases, there was no long-term follow-up, so post-discharge complications may be undercounted, the socioeconomic profile of inpatients may not represent the wider region, ambulatory cases were excluded, and important unmeasured factors (e.g., lifestyle, education, environmental exposures) were not incorporated.

## Conclusions

Atrial fibrillation is the most common clinical arrhythmia and carries substantial morbidity and mortality, underscoring the need for rigorous, tailored care. Our study at Hassan II hospital in Agadir examined the epidemiologic, clinical, diagnostic, etiologic, therapeutic, and outcome features of AF in our setting. AF predominantly affected older adults with cardiovascular risk factors and underlying heart disease; dyspnea with signs of heart failure was the leading complaint. ECG remained the cornerstone for diagnosis and detection of electrical abnormalities, while transthoracic echocardiography, chest radiography, and laboratory testing were essential for identifying causes and guiding therapy. Valvular etiologies persisted among younger patients, whereas non-valvular AF prevailed in older adults. Thromboembolic and bleeding risk stratification directed anticoagulation decisions; in practice, direct oral anticoagulants were underused, with vitamin K antagonists favored. Permanent AF was more frequent and typically managed with rate rather than rhythm control. These findings highlight the need to strengthen prevention, expand access to newer treatments, and develop protocols adapted to local realities, while encouraging future research to refine care and reduce the regional burden of AF through integrated prevention, early diagnosis, and personalized management.
